# Evaluation of CBR of Graded Crushed Stone of Flexible Base Structural Layer Based on Discrete Element Model

**DOI:** 10.3390/ma16010363

**Published:** 2022-12-30

**Authors:** Zhaoguang Hu, Heng Liu, Weiguang Zhang, Tianqing Hei, Xunhao Ding, Zezhen Dong

**Affiliations:** 1 China Road and Bridge Corporation, Dongcheng District, Beijing 100011, China; 2 School of Foreign Languages, Jiangsu Open University, Nanjing 210036, China; 3 School of Transportation, Southeast University, Nanjing 211189, China

**Keywords:** graded crushed stone, gradation, the discrete element method, the CBR test

## Abstract

In order to study the mechanical properties of graded crushed stone, the discrete element method is used to simulate the CBR test of graded crushed stone. Aiming at the composition structure of graded crushed stone material, the PFC3D simulation software is used to construct the test model, and the process of constructing the virtual specimen model of the graded crushed stone discrete element model is discussed in detail. A servo mechanism is used to control the speed of the wall in the software, so as to control the virtual confining pressure imposed on graded crushed stone by the wall and simulate the real CBR test environment. The micro-parameter calibration of the virtual test is carried out by comparing the indoor and virtual CBR specimens of a single particle size specimen and three groups of graded crushed stone specimens. The comparison result shows that the stress–strain characteristics of the graded crushed rock obtained by the discrete element simulation during the uniaxial penetration process have a high degree of similarity, which can verify the accuracy of the model establishment. With the increase in the penetration depth, the penetration force of the aggregates of various particle sizes gradually increases, and the penetration force and the penetration depth are basically linear, and when the particle size is greater than 9.5 mm, the increase in particle size has little effect on the CBR test results. Under the certain conditions, the contact stiffness of graded crushed stone particles with particle sizes of 4.75 mm, 9.5 mm, 13.2 mm, 16 mm, and 19 mm should be 0.88 × 10^7^ (N/m), 0.98 × 10^7^ (N/m), 1.10 × 10^7^ (N/m), 1.25 × 10^7^ (N/m), and 2.05 × 10^7^ (N/m), respectively. The recommended value of the contact stiffness of the small spherical particles increases with the increase in the particle size. This model can provide a basis for studying the micromechanical state of graded crushed stone and physical mechanics tests.

## 1. Introduction

The application of graded crushed stone to the pavement structure base has many advantages, such as reducing reflection cracks and prolonging the service life of the pavement [1]. In order to obtain high-quality graded crushed stone, it is necessary to evaluate its performance with the help of the California bearing ratio CBR, resilience modulus, water permeability coefficient, and other indicators [2]. Chen [3] obtained from the actual test that the modified vibration molding method is in good agreement with the compaction degree of the actual project. Based on the indoor test of the modified vibration molding method, Chen proposed a series of performance design indexes for the design of graded crushed stone. Through a large number of repeated triaxial compression tests, Caroline [4] summarized some design rules of graded crushed stone grading for graded crushed stone design. Jiang [5] proposed a strength prediction equation for cement-graded crushed stone to calculate the strength development law of cement-graded crushed stone. Gustavo [6] presented a methodology for the experimental characterization of graded crushed stone, with a focus on mechanistic design. Xiao [7] investigated the effect of quality of graded crushed stone on conventional flexible pavement performance in Minnesota through a mechanistic–empirical pavement design approach. Zhang [8] proposed a random generation model to simulate the shear behavior of graded crushed stone.

Usually, researchers carry out virtual simulation experiments of various road materials based on particle discrete element software [9]. There are not only asphalt mixture simulations based on parametric shape and size and grading design [10], but also virtual numerical simulation tests of graded crushed stone for road bases.

Shen [11] evaluated the influence of skeleton stacking on the mechanical properties of the mixture by calculating the contact force chain and the average contact force. Chen [12] used the method of overlapping multiple spheres to characterize the irregular shape of the coarse aggregate, and quantitatively analyzed the effect of each particle size aggregate on the aggregate gap ratio, the number of contact points between aggregates, the contact force, and other parameters in the mixture after step-by-step filling. 

Peng [13] established a two-dimensional meso model, and then studied the California bearing ratio test of graded crushed stone. Jiang [14] constructed a biaxial numerical test method for graded crushed stone to analyze the variation law of each index. Both Jiang and Zhang [9,15] independently established a numerical model for dynamic triaxial testing of graded crushed stone, which is used to simulate real tests, thereby reducing the number of real triaxial test models. Xiao [16] established a three-dimensional discrete element model considering the irregular shape of real particles to reveal the shear strength mechanism and evolution characteristics of skeleton hollow graded broken stone.

Cao [17] conducted a virtual triaxial test of graded crushed stone and analyzed the stress–strain relationship of the skeleton structure of graded crushed stone and the influence of gradation composition and skeleton porosity on the stress–strain curve. 

Zhang [18] proposed a method to generate graded broken stone discrete element test pieces, which is used to simulate the laboratory test of the rebound modulus of graded broken stone.

Liu [19] obtained the basic law that the peak deviatoric stress of graded gravel increases with the increase in confining pressure through static triaxial virtual testing of graded gravel. The influence of grading on the elastic modulus was analyzed, and the nonlinear elastic modulus constitutive model equation was obtained by fitting.

Within the gradation range limited by the graded crushed stone base specification, the performance of the graded crushed stone is quite different [20]. This can be seen from the different CBR values of different grades of crushed stone. In order to obtain the grading of graded crushed stone with high CBR value, it is necessary to combine certain practical tests within the specified range, which will lead to a waste of more materials and time. Therefore, in order to solve these problems, it is necessary to design a numerical model to simulate a CBR test. At present, through the establishment of a virtual model, the research on the micro-effects of graded crushed stone became mature, but there is a lack of research on the macro-performance of graded crushed stone, such as CBR. This paper uses PFC3D to simulate the laboratory test of graded crushed stone, and builds a simulation model of the mechanical properties of graded crushed stone through the selection of logic language, the selection of contact model, the generation of graded particles, the realization of loading method, and the definition of material microscopic parameters. Through the comparison of a single-particle size specimen and three groups of graded crushed stone specimens with virtual CBR specimens, the micro-parameter calibration of the virtual test is carried out. The simulation analysis of this model can explain the relationship between the mechanical response state, mechanical parameters, and mechanical properties of graded crushed stone from a microscopic point of view, and provide a basis for subsequent physical and mechanical tests of graded crushed stone.

## 2. Basic Principles of Discrete Element Method

### 2.1. Force–Displacement Equation and Motion Relationship

The calculation of discrete element software is based on the most basic stress–strain relationship and the force–motion relationship with Newton’s second law as the core. In each time step (small enough), the force state between particles changes, and physical quantities such as velocity, displacement, and strain are updated accordingly. These are all premised on the contact between the media and are calculated from the normal stiffness, tangential stiffness, contact overlap, and other parameters as follows:

(1) $$F_{i}^{n}=k_{i}^{n}x_{i}^{n}n_{i}$$

(2) $$F_{i}^{s}=-k_{i}^{s}\Delta x_{i}^{s}$$

(3) $$\Delta x_{i}^{s}=V_{i}^{s}\Delta t$$

(4) $$V_{i}^{s}=V_{i}-V_{i}^{n}$$

Among them, $F_{n}^{i}$ and $F_{i}^{s}$ are the normal and tangential components of the contact force at the contact point *i*, and the latter is calculated in increments; $k_{i}^{n}$ and $k_{i}^{s}$ are normal contact stiffness coefficients and tangential contact stiffness coefficients; $x_{i}^{n}$ and Δ$x_{i}^{s}$ are the contact overlap and contact displacement increment at the contact point; *n_i_* is the unit normal vector of the contact surface; *V_i_*, $V_{i}^{n}$, vs. *_i_* are the contact point velocity and the normal and tangential components; Δ*t* is the time step. 

For a single particle, its motion state is calculated from the angular velocity and linear velocity, while for the entire structure or particle system, its velocity is calculated by Newton’s second law, which is expressed as the following functional relationship:

(5) $$w=f_{1}\left(a\right)$$

(6) $$v=f_{2}\left(a\right)$$

(7) $$V_{i}=f\left(w,v\right)$$

Among them, *w* and *v* are the angular velocity and linear velocity of the particle system; a is the acceleration of the particle system.

### 2.2. Contact Constitutive Model

Contacts in discrete elements can be divided into two types: “ball-ball” contact and “ball-wall” contact. The contact constitutive model is used to define the contact action. The contact constitutive model during simulation in PFC3D software (PFC3D-5.1, Itasca, IL US) mainly includes: linear contact stiffness model and slip model.

The linear contact stiffness model is a model proposed by Cundall in 1979 to characterize the interaction between aggregate particle units. The contact stiffness includes the normal stiffness coefficient *k^n^* and the tangential stiffness coefficient $k^{s}$, and its value is determined by the stiffness coefficients of the entities A and B at both ends of the contact, as shown in Equations (8) and (9).

(8) $$k^{n}=\frac{k_{n}^{A}k_{n}^{B}}{k_{n}^{A}+k_{n}^{B}}$$

(9) $$k^{s}=\frac{k_{s}^{A}k_{s}^{B}}{k_{s}^{A}+k_{s}^{B}}$$

Among them, $k_{n}^{A}$ and $k_{n}^{B}$ are the normal contact stiffnesses of the two-end contact entities; and $k_{s}^{A}$ and $k_{s}^{B}$ are the tangential contact stiffnesses of the contact two-end entities.

The slip model is a contact action model for judging whether the entities at the two ends of the contact slip, and it is mainly calculated based on the normal contact force, the tangential contact force, and the friction coefficient. The formula for calculating the maximum tangential contact force that the contact point can bear is shown in Equation (10). When |$F_{i}^{s\;}$|> $F_{max}^{s\;}$, relative sliding occurs between the two contact entities.

(10) $$F_{max}^{s\;}=\mu \left|F_{i}^{n}\right|$$

Among them, $F_{max}^{s}$ is the maximum tangential contact force that the contact point can bear; μ is the friction coefficient; and $F_{i}^{n\;}$ is the normal contact force.

## 3. Basic Principles of Discrete Element Method

### 3.1. Virtual Mold Setting

**Figure 1:**
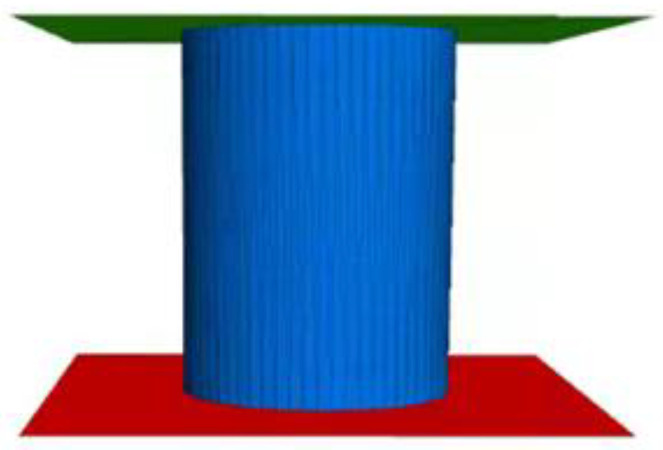
Schematic diagram of CBR test mold.

Use the “generate” command to generate aggregates with a specific gradation, and determine the number of generated particles and the number of attempts to ensure that particles do not overlap. In order to be similar to the form and size of the graded crushed stone specimen in the actual indoor test, the virtual mold adopts a cylindrical mold, which is composed of a cylindrical wall, an upper plane wall and a lower plane wall, as shown in Figure 1. Among them, the cylindrical wall is formed by splicing triangular plane walls. The more the number of triangular walls, the closer the cylindrical wall is to a smooth cylindrical surface, and the higher the calculation accuracy is. At the same time, the increase in the number will reduce the calculation efficiency. The number and precision of the triangular walls in this paper are set by the resolution parameter.

The diameter of the cylindrical specimen is 150 mm, which is consistent with the CBR test of the intermediate grade crushed stone in the “Geotechnical Test Method Standard GB/T 50123-2019” (MOHURD 2019) [21]. Compared with the indoor test, the random generation of aggregate particles in the virtual test requires sufficient space, so the height of the virtual mold is set to 1.6 times the height of the target specimen, and the height of the CBR test mold is set to 194 mm.

### 3.2. Specimen Setting of Graded Particles

The height, radius, initial void ratio, and particle size of the test piece are known, and on this basis, a digital test piece of graded crushed stone is generated. In order to better fit the actual specimen forming process, after the virtual mold is formed, the graded crushed stone specimen with the specified gradation is formed in three layers, and the thickness of each layer is equal, which is 1/3 of the final height of the specimen.

#### 3.2.1. Calculation of The Number of Particles Corresponding to Gradation

In the actual indoor test, the gradation control is realized by calculating and weighing the specified mass of aggregates with different particle size ranges. In this paper, in the PFC software, the number of particles is also calculated according to the principle of equal mass. During the simulation, the mass relationship is converted into a volume relationship by assigning different densities to particles of different sizes. Since the graded crushed stone specimens are made of the same material, the aggregates have the same density.

According to the percentage passing of each sieve hole in the gradation, the total volume of the aggregate is allocated to each grade of aggregate according to the percentage retained, and then according to the aggregate density and the corresponding particle size to calculate the number of particles needed to generate each grade of aggregate. The formula for calculating the number of particles is shown in Equations (14)–(17).

(11) $$V=V_{0}\left(1-poros\right)$$

(12) $$V_{i}=V\alpha_{i}$$

(13) $$\alpha_{i}=P_{i+1}-P_{i}$$

(14) $$N_{i}=\frac{V_{i}}{v_{i}}$$

(15) $$v_{i}=\frac{4\pi R_{ave}^{3}}{3}$$

(16) $$R_{ave}=\frac{d_{i+1}+d_{i}}{4}$$

In the formula, *V*_0_, *V*, *V_i_*, and *v_i_*—the volume of the specimen, the total volume of the specimen particles (excluding voids), the total volume of the i-th grade particles, and the volume of a single particle of the i-th particle. *α_i_, p_i+_*_1_*,* and *p_i_* are the volume fraction of the i-th grade particles to the specimen, respectively, and the sieve pass rate of the i + 1-th grade and the i-th grade; N_i_ is the number of i-th grade particles; poros is the porosity of the specimen; *R_ave_*, *d_i+1_*, and d_i_ are the average particle size of the i-th grade particles, respectively (to simplify the calculation, it is regarded as the average value of the maximum and minimum particle size of this grade of particles), in summary:

(17) $$N_{i}=\frac{\pi \cdot 0.12\cdot 0.15^{2}\left(1-\mathrm{poros}\right)\Delta P}{4}\cdot {\left(\frac{4\pi \left(\frac{d_{i+1}+d_{i}}{4}\right)}{3}\right)}^{-1}$$

#### 3.2.2. Generation Process of Target Graded Crushed Stone Specimens

In order to obtain the uniform compaction effect of graded crushed stone, the model is compacted three times in the PFC software, and the number of aggregate particles generated each time is one third of the target particle number of each grade. The first (bottommost) layer of aggregate particles is formed first. The newly generated particles are in a loose state and have a large void ratio, occupying the space from the bottom of the mold to 1/2 the height of the mold. When generating particles, according to the actual situation when generating particles, specify the number of attempts through the tries command.

In this paper, vibration is used for particle compaction to ensure more reasonable distribution of aggregate particles of different sizes and closer contact [22,23]. The particles are given a gravity of 9.8 m/s^2^. The frequency of 5 Hz was selected for vibration, and the vibration velocities of 5 cm/s and 1 cm/s were firstly applied in the z and x directions, and then the vibration velocities of 5 cm/s and 1 cm/s were applied in the z and y directions to realize the redistribution of the pellets. After the vibration is completed, a wall parallel to the mold base is generated at the highest point of the particles as a loading plate, and the loading plate is given a downward speed to form a load on the aggregate particles, simulating the actual graded crushed stone compaction process, until the aggregate particles are pressed to the specified height. After the compaction is completed, the loading plate is given a slight upward speed to move it up slowly until it is not in contact with the aggregate particles and is then removed to provide space for the second layer of compaction.

**Figure 2:**
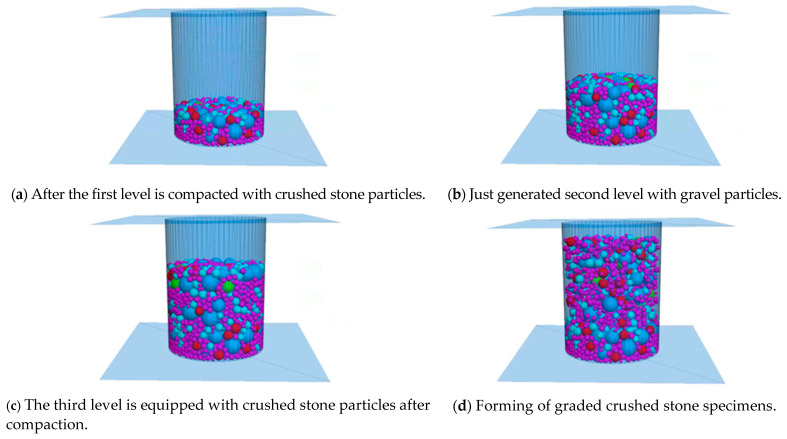
Graded gravel specimen forming process.

Repeat the above steps to generate the second layer and the third layer of aggregate particles. The generation range of the second layer of aggregate particles is from the top of the first layer of aggregate to 0.75 times the height of the mold, and the load plate presses it to 2/3 of the height of the test piece. The generation range of the third layer aggregate particles is from the top of the second layer aggregate to the height of the mold, and the load plate presses it to the height of the target specimen. After the above steps, the virtual test piece of the target cylinder is finally formed, and the specific molding process of the test piece is shown in Figure 2.

### 3.3. Setting of Wall Speed and Confining Pressure

The speed of the wall is controlled by a servo mechanism to ensure that the wall exerts a specific pressure, which is used to simulate the compaction process of graded crushed stone [24]. The confining pressure is realized by two parameters: target confining pressure and allowable error. Servo control the wall speed and calculate the stress on the wall in real time and compare it with the required applied stress. If the two errors are less than the allowable error parameter, it indicates that the confining pressure was achieved. If the two errors exceed the allowable error parameter, the cycle will continue until the requirements are met.

The above comparison of wall velocity and stress error is carried out continuously during the virtual test. Assuming that there is a corresponding relationship between the wall velocity and the load applied to the wall, the wall velocity can be expressed as:

(18) $${\dot{u}}^{\left(wall\right)}=G\left(\sigma_{measured}-\sigma_{required}\right)=G\Delta \sigma$$

Among them, ${\dot{u}}^{\left(wall\right)}$ is the wall velocity; *σ_measured_* is the measured stress value; σ_required_ is the predetermined stress value; and Δ*σ* is the stress difference. G is the parameter of the servo mechanism, and the dimension is dim G = L^2^M^−1^T, which is used to control the speed of the wall.

The wall and the sphere are in contact with each other, and the wall moves in the direction of the sphere to receive the resistance force from the sphere, and the sphere receives the reaction force from the wall at the same time. In the time step Δ*t*, the reaction force increment, of Δ*F^(wall)^* of the wall acting on the sphere is:

(19) $$\Delta F^{\left(wall\right)}=k_{n}^{\left(average\right)}N_{c}{\dot{u}}^{\left(wall\right)}\Delta t$$

Among them, Δ*F^(wall)^* is the increment of the force on the wall; $k_{n}^{\left(average\right)}$ is the average stiffness of the contact between the wall and the sphere; *N_c_* is the number of contacts between the wall and the sphere; ${\dot{u}}^{\left(wall\right)}$ is the wall velocity; and Δ*t* is the time step.

According to the stress calculation formula, the calculation formula of the stress increment applied by the wall is:

(20) $$\Delta \sigma^{\left(wall\right)}=\frac{\Delta F^{\left(wall\right)}}{A}=\frac{k_{n}^{average}N_{c}{\dot{u}}^{\left(wall\right)}\Delta t}{A}$$

Among them, Δ*σ^(wall)^* is the stress increment applied by the wall; and A is the area of the wall where the load is applied.

In order to ensure that the expected load is applied to the wall, the value of Δ*σ^(wall)^* should be strictly controlled. In this paper, the accuracy of the stress applied to the wall is controlled by taking the safety factor α(α < 1), that is, the increment Δ*σ^(wall)^* of the stress applied to the wall should be less than the difference Δ*σ* between the actual measured stress of the wall and the predetermined stress of the wall:

(21) $$\left|\Delta \sigma^{\left(wall\right)}\right|=\alpha \left|\Delta \sigma \right|$$

Substitute Equations (18) and (20) into Equation (21) to get:

(22) $$\alpha \left|\Delta \sigma \right|=\frac{k_{n}^{\left(average\right)}N_{c}G\left|\Delta \sigma \right|\Delta t}{A}$$

Eliminate |Δσ| in Equation (22), we get:

(23) $$G=\frac{\alpha A}{k_{n}^{\left(average\right)}N_{c}\Delta t}$$

### 3.4. Simulation of CBR Loading Conditions

**Figure 3:**
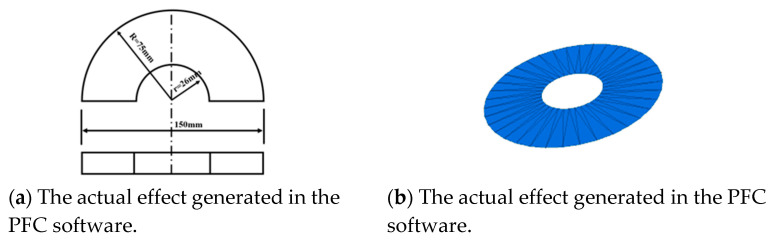
Schematic diagram of CBR test circle load block.

During the CBR test, four pairs of semi-circular ring load blocks with a diameter of 150 mm and a center hole diameter of 52 mm should be placed above the specimen, and each pair weighs 1.25 kg, as shown in Figure 3a. Use MATLAB (2020a, MathWorks, Natick, MA, USA) software to generate circles with a diameter of 150 mm and a diameter of 52 mm on the x-y plane, divide the circle into 36 equal parts, and derive the coordinates of each bisected point. In the PFC software, the wall create command is used to input the coordinates of the above-mentioned equal points, and the generated small triangles finally form a circular wall, as shown in Figure 3b. After the annular load plate is generated, the above-mentioned servo method is used to simulate the gravity effect of the load block based on the wall stress.

**Figure 4:**
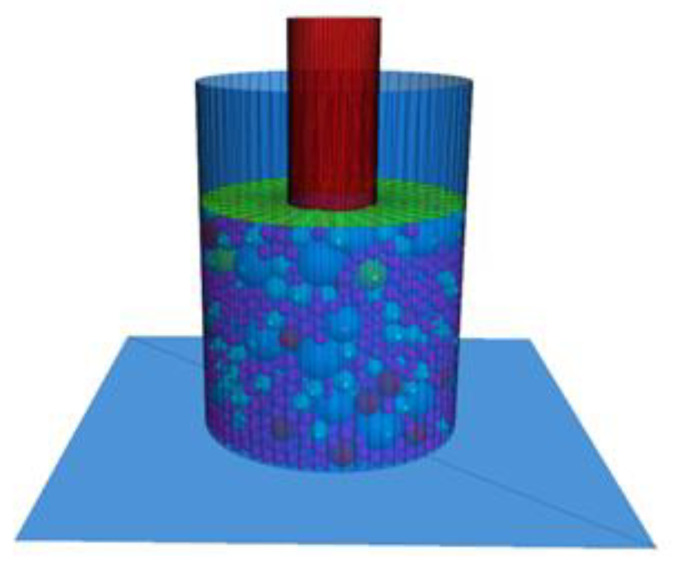
Virtual CBR test loading schematic.

A small cylinder with a diameter of 5 cm and a height of 5 cm and a disc with a diameter of 5 cm were combined as the indenter, and the initial position was located above the center of the specimen, as shown in Figure 4. Considering the actual running speed and calculation time, the penetration speed of the virtual test is set to 2 mm/min and adjusted according to the subsequent loading conditions. After loading, with the monitored penetration depth as the control condition, when it reaches 8 mm, the loading stops.

## 4. Calibration of microscopic parameters

### 4.1. Indoor CBR Test

Carrying out indoor CBR experiments can provide data support for determining the micro-parameter correction of discrete element CBR in virtual experiments.

#### 4.1.1. Material selection and specimen grading

Taking into account factors such as material and cost, limestone is selected as the research object. The following test pieces were formed in the laboratory test:

(1) Prepare coarse aggregate specimens with a single particle size, including 4.75 mm, 9.5 mm, 13.2 mm, 16 mm, and 19 mm;

(2) The middle value and upper and lower limits of grading type G-A-4 in the “Highway Pavement Base Construction Technical Specifications” (JTG/T F20-2015) [25] shall be selected, the fine aggregate part of the grading type shall be removed, and the proportion of the part above 4.75 mm shall be expanded. The gradation of the indoor test specimens is shown in Table 1.

#### 4.1.2. Forming Steps of The Test Piece

**Figure 5:**
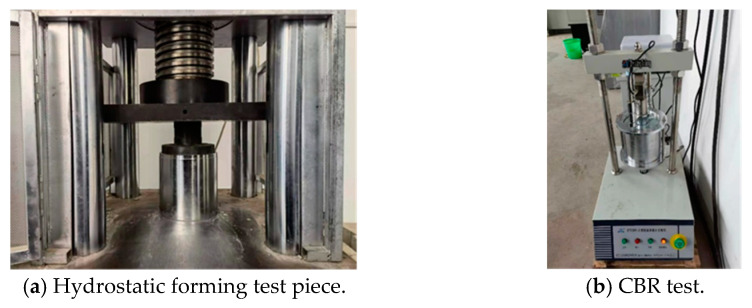
Indoor CBR test procedure.

In this study, the static compaction method was used to form graded crushed stone specimens with a size of 150 mm in diameter and 120 mm in height. Before static pressing, put the crushed stone into the test tube in three steps, and strike 10 times with a small hammer each time to make the aggregate initially compact. After putting all the crushed stone into the test tube, it was placed under a press to form under static pressure, with a load of 15 KN and a static pressure of 10 s, as shown in Figure 5a.

#### 4.1.3. Indoor CBR Test

**Figure 6:**
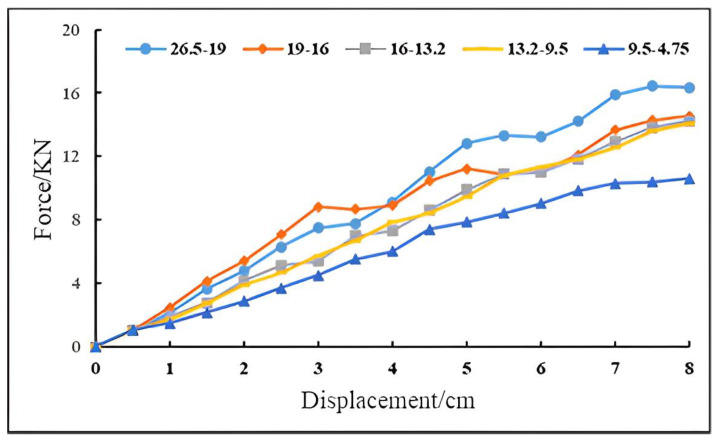
Indoor CBR test results.

The formed specimen was placed on the digital CBR tester, and four pairs of semicircular load blocks were placed on the surface of the specimen. Adjust the height of the lifting platform on the bottom of the test piece and the position of the sensor so that the sensor is just placed on the edge of the test tube at the beginning of the test. Set the loading speed of the loading indenter to 1 mm/s, set the loading stop condition as the penetration depth reaching 8 mm, and carry out the CBR test (see Figure 5b). Three indoor tests are repeated for each single particle size, and the final results are taken as the average of the three tests. After the test, the instrument will automatically output the relationship between the loading indenter pressure and the penetration depth (with the penetration depth of 0.5 mm as the interval) to draw the stress–displacement curve. The indoor CBR test results are shown in Figure 6.

Observing the test curve, it can be seen that as the penetration depth (displacement) increases, the penetration force of each grade of particle size aggregate increases gradually, and the penetration force and penetration depth are basically linear. This is because as the penetration progresses, the gaps between the graded crushed stone particles gradually decrease, and the entrapment effect is strengthened. To obtain the same displacement, the force required by the load head must increase [26]. Among the coarse aggregates with a single particle size in the fifth grade, except for the 4.75 mm particle size aggregate, which has a slightly smaller penetration force, the penetration force of other particle sizes is not much different. The penetration force of the 19 mm particle size aggregate is slightly larger than that of other particle sizes when the penetration depth is greater than 4 mm. Therefore, it is not recommended to blindly pursue large-sized aggregate skeletons in order to increase the load bearing capacity when selecting the gradation of crushed stone.

### 4.2. Calibration Results of Microscopic Parameters

In order to make the virtual specimen of discrete element model simulate the actual test situation, the contact stiffness, friction coefficient and other microscopic parameters are calibrated by comparing the penetration force penetration depth curves of virtual CBR test and indoor CBR test. The number of virtual tests is not certain. The error between the virtual test results and the indoor test results is controlled within 5% through multiple iterations, so as to calibrate the contact stiffness, friction coefficient, and other microscopic parameters of the discrete element entities (walls and balls). Combined with studies [27,28], it is found that among the microscopic parameters of normal phase stiffness, tangential stiffness, and friction coefficient between the sphere and the wall, the normal contact stiffness of the sphere has the greatest influence on the penetration force. Therefore, in this paper, the normal stiffness and tangential stiffness of the wall are set to 1 × 10^10^ (N/m), the friction coefficient is 0.7, and the spherical friction coefficient is 0.5. By changing the normal and tangential stiffness of the sphere (general normal stiffness = tangential stiffness), the virtual test results closest to the laboratory test results are obtained. The selection of parameters is shown in Table 2.

**Figure 7:**
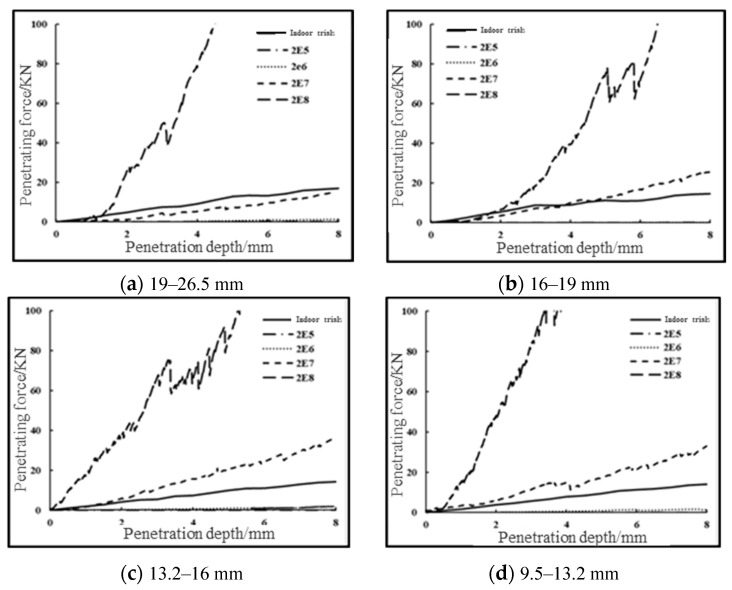

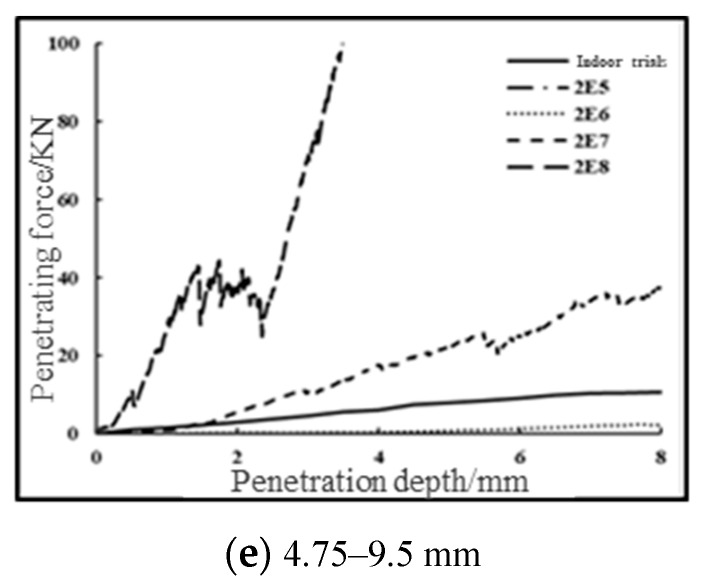
Comparison of indoor and virtual CBR test results for single particle size specimens with different microscopic parameters.

The virtual uniaxial penetration tests with 4.75 mm, 9.5 mm, 13.2 mm, 16 mm, and 19 mm grain sizes were carried out according to the steps of virtual CBR test model construction using different spherical method/tangential stiffness in the table, and the comparison results with the indoor tests are shown in Figure 7.

It can be seen that the magnitude of the penetration force obtained from the virtual test is positively correlated with the ball stiffness, and when the ball stiffness is 2 × 10^8^ (N/m), the penetration force obtained from the simulation is much larger than the actual value, which is 15–25 times that of the actual value, and is undesirable. When the sphere stiffness is 2 × 10^5^ (N/m) and 2 × 10^6^ (N/m), the simulated penetration force is much smaller than the actual value, which is about 1/1000 and 1/100 of the actual value, which is not desirable.

When the sphere stiffness is 2 × 10^7^ (N/m), the simulated penetration force is slightly smaller than that of the indoor test for 19 mm particle size graded aggregates; for 16 mm particle size graded aggregates, the simulated penetration force is slightly larger than that of the indoor test; for 13.2 mm, 9.5 mm, and 4.75 mm particle size graded aggregates, the simulated penetration force is larger than that of the indoor test. The simulated penetration force for 13.2 mm, 9.5 mm, and 4.75 mm graded aggregates is greater than the indoor test results.

Therefore, based on the results of the above analysis, the microscopic parameters were further optimized. For the graded crushed stone with the particle size of 19mm and 16mm, the values larger than and smaller than 2 × 10^7^ shall be selected for further verification, and 0.05 × 10^7^ shall be taken as the change step of the stiffness of the small ball each time. For the other three groups of particle size test pieces, take 2 × 10^6^ and 2 × 10^7^ as the sphere stiffness value interval, and continuously use the dichotomy method to approximate the final value of the sphere stiffness. Compare the simulation results with the indoor test results, when the difference between the two is less than 5% of the indoor test results, the parameter optimization is stopped, and the small ball stiffness is considered as the final value of small ball stiffness for the corresponding particle size at this time.

**Figure 8:**
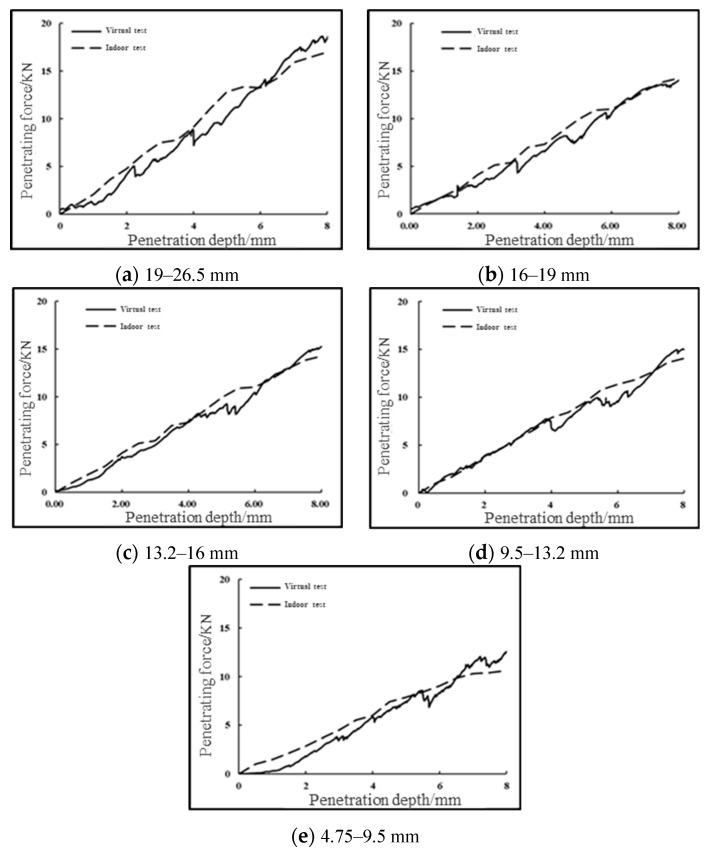
Final comparison of indoor and virtual CBR test results for single particle size specimens.

The optimization results of the microscopic parameters for different particle sizes of graded aggregates are shown in Table 3, and the comparison between the virtual CBR test results of graded aggregates using the microscopic parameters in the table and the indoor CBR test results is shown in Figure 8, and the errors are within the permissible range. It can be seen that the results of the virtual test and the indoor test are roughly consistent. There are two reasons for the deviation. Firstly, that during the indoor test, the data are read every 0.5 mm and are connected into a curve due to the equipment limitation, while in the virtual test, the results are recorded every step, which can be regarded as an almost continuous curve. Secondly, the position of each particle with the same grading is different, which leads to the lateral slip of gravel particles at different positions during the penetration process, resulting in up and down fluctuations at different positions of the curve.

**Figure 9:**
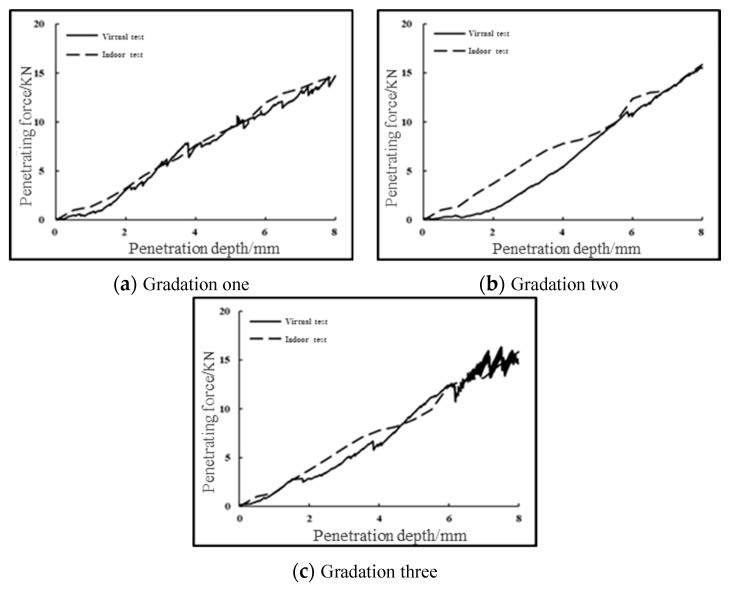
Comparison of indoor and virtual CBR test results for the G-A-4 three-group grading.

In order to further verify the correctness of the calibrated microscopic parameters, three groups of gradations of the upper, middle, and lower limits of G-A-4 are obtained in “Technical Rules for Construction of Highway Pavement Base” (JTG/T F20-2015) (MOT 2015). The fine aggregate part is then removed. Next, the coarse aggregate part is enlarged in equal proportion, and the virtual CBR test is carried out using the microscopic parameters shown in the Table 3, and the results are compared with the indoor test results. For the penetration force–penetration depth curve of the virtual CBR test and the indoor CBR test, the error of the penetration forces of gradation one, gradation two, and gradation three under different penetration depths shall not exceed 5%, 15%, and 10%, respectively. The results are shown in Figure 9.

According to the comparison between the virtual test and the indoor test, it can be seen that the numerical test results of CBR test can be well matched with the indoor test results. The correctness of the calibrated micro-parameters in Table 3 is further verified, so the stress–strain characteristics during the uniaxial penetration of graded crushed stone can be predicted relatively quickly through indoor simulation tests.

## 5. Conclusions

In this paper, the virtual model of graded crushed stone is improved from the interaction of micro-graded crushed stone particles to the performance of macro-graded crushed stone, and the performance prediction model of two-dimensional graded crushed stone CBR is extended to the performance prediction model of three-dimensional graded crushed stone CBR, which provides a basis for the physical and mechanical tests of graded crushed stone in the future.

The discrete element software PFC3D is used to simulate the main calculation principle, specimen forming method, and loading method of the test of graded crushed stone, and combined with the indoor CBR test, the microscopic parameters of the virtual test are calibrated. The main research conclusions are as follows:

(1) According to the non-bonding characteristics of graded crushed stone, when simulating in PFC3D software, the linear stiffness model and the slip model are used. When the specimen is simulated and formed, enough space is reserved for the compaction process, and the height of the specimen mold wall is 1.6 times the target height of the specimen. The aggregate particles are put in three times. After each time, gravity should be applied first, then vibration should be applied, and then a pressure plate should be formed for compaction. After compaction, it is recommended to slowly remove the pressure plate to prevent large disturbance of the particles.

(2) The pressure cannot be directly applied in the PFC3D software, so the servo mechanism is used to apply a constant load through the relationship between the servo parameter G, speed, and stress. The loading of the CBR test is a strain-controlled mode, which is accomplished by imparting a constant speed to the indenter.

(3) As the penetration depth (displacement) increases, the penetration force of each grade of particle size aggregate gradually increases, and the penetration force and penetration depth are basically linear. According to the relationship between the penetration force of coarse aggregates with a single particle size of five grades, when the particle size is larger than 9.5 mm, the increase in particle size has little effect on the CBR test results. Therefore, it is not necessary to blindly pursue a large particle size in order to obtain a larger bearing capacity.

(4) Combined with the indoor CBR test, the micro-parameter calibration method of the discrete element simulation test of graded crushed stone is proposed. Among the microscopic parameters, the contact stiffness of the spherical particles has the greatest influence on the simulation results. The calibration found that when the normal and tangential contact stiffness of the wall is 1e10, the friction coefficient of the wall is 0.7, and the friction coefficient of the sphere is 0.5, the contact stiffness of graded crushed stone particles with particle sizes of 4.75 mm, 9.5 mm, 13.2 mm, 16 mm, and 19 mm should be 0.88 × 10^7^, 0.98 × 10^7^, 1.10 × 10^7^, 1.25 × 10^7^, and 2.05 × 10^7^, respectively.

## Figures and Tables

**Table 1:** Specimen grading for indoor test.

Gradation Types	Mass Percentage (%) Passing Through the Following Square Mesh Sieve (mm)					
	26.5	19	16	13.2	9.5	4.75
Gradation one	100	82	70	60	40	0
Gradation two	100	75	63	52	34	0
Gradation three	100	70	57	44	27	0

**Table 2:** Preliminary table of micro-parameter values.

	Sphere	Wall
Normal stiffness (N/m)	2 × 10^5^/2 × 10^6^/2 × 10^7^/2 × 10^8^	1 × 10^10^
Tangential stiffness (N/m)	2 × 10^5^/2 × 10^6^/2 × 10^7^/2 × 10^8^	1 × 10^10^
Friction coefficient	0.5	0.7

**Table 3:** Micro-parameter values.

	Sphere					Wall
Aggregate Size (mm)	4.75	9.5	13.2	16	19	All
Normal stiffness (N/m)	0.88 × 10^7^	0.98 × 10^7^	1.10 × 10^7^	1.25 × 10^7^	2.05 × 10^7^	1 × 10^10^
Tangential stiffness (N/m)	0.88 × 10^7^	0.98 × 10^7^	1.10 × 10^7^	1.25 × 10^7^	2.05 × 10^7^	1 × 10^10^
Friction coefficient	0.5					0.7

## Data Availability

Some or all data, models, or code that support the findings of this study are available from the corresponding author upon reasonable request, including specific dimensional stiffness.
